# Supplementary material for: Modelling the economic burden of SARS-CoV-2 infection in health care workers in four countries
Source: Nat Commun. 2023 May 16;14:2791. doi: 10.1038/s41467-023-38477-7 (PMC10185455; doi:10.1038/s41467-023-38477-7)
Supplement: Supplementary file 1 — Supplementary Information [file 41467_2023_38477_MOESM1_ESM.pdf]

Supplementary Information

Modelling the economic burden of SARS-CoV-2 infection in health care workers in four countries

Huihui Wang[1], Wu Zeng[2*], Kenneth Munge Kabubei[3], Jennifer Rasanathan[4], Jacob Kazungu[5], Sandile Ginindza,[6] Sifiso Mtshali[7], Luis E. Salinas[8], Amanda McClelland[9], Marine Buissonniere[9], Christopher T. Lee[9], Jane Chuma[3], Jeremy Veillard[8], Thulani Matsebula[10], Mickey Chopra[1]

1. World Bank, Washington, DC, USA
2. Department of Global Health, Georgetown University, Washington, DC, USA
3. World Bank Kenya Office, Nairobi, Kenya
4. Independent Consultant, Geneva, Switzerland
5. Health Economics Research Unit, KEMRI Welcome Trust Research Program, Nairobi, Kenya
6. Pact, Mbabane, Eswatini
7. Public Health Medicine Department, University of KwaZulu-Natal, Durban, South Africa
8. World Bank Colombia Office, Bogota, Colombia
9. Resolve to Save Lives, New York, NY, USA
10. World Bank South Africa Office, Pretoria, South Africa

***Corresponding author**
Wu Zeng, Department of Global Health, Georgetown University. 3700 Reservoir Rd NW, Washington DC. Email: wz192@georgetown.edu

Table of Contents

Supplementary Table 1. COVID-19 cases and deaths among HCWs and the general population, by study site 3

Supplementary Table 2. Total economic burden and economic cost per SARS-CoV-2 infection among HCWs in international dollars (I$) 4

Supplementary Table 3. Country profiles of study sites 5

Supplementary Table 4. Sources of key parameters for Kenya 6

Supplementary Table 5. Sources of key parameters for Eswatini 7

Supplementary Table 6. Sources of key parameters for Colombia 8

Supplementary Table 7. Sources of key parameters for Western Cape and KwaZulu of South Africa ... 9

Supplementary Table 8. Sources of parameters applied to all study sites 11

Supplementary Fig. 1. Tornado graphs representing sensitivity analyses 12

Supplementary Methods. Estimation of exposure rate and odds ratio for secondary infection 13

Supplementary Table 1. COVID-19 cases and deaths among HCWs and the general population, by study site

	Kenya	Eswatini	Colombia	SA-WC	SA-KZN
**Background indicators**					
Population size (2019, in millions)	52.57	1.15	50.34	7.01	11.53
Total number of HCWs (2019)	176,662	7,726	838,651	35,317	68,862
Number of HCWs per 1,000 (2019)	3.40	6.70	16.70	5.00	6.00
**COVID-19 cases (March 1, 2020-Feb 28, 2021)**					
Number of COVID-19 cases in the general population	105,148	17,911	2,250,173	297,043	341,336
Number of COVID-19 cases in HCWs	3,400	464	42,142	10,111	16,299
Share of COVID-19 cases that occurred in HCWs	3.20%	2.53%	1.86%	3.37%	4.66%
Incidence of COVID-19 in the general population (per 1000 population)	2.0	15.6	44.7	42.4	29.6
Incidence of COVID-19 in HCWs (per 1000 population)	19.2	60.1	50.2	286.3	236.7
**COVID deaths (March 1, 2020-Feb 28, 2021)**					
Number of COVID-19 deaths in the general population	1,853	670	60,511	11,881	10,535
Number of COVID-19 deaths in HCWs	33	10	196	108	386
Share of deaths that occurred in HCWs (%)	1.78%	1.49%	0.32%	0.91%	3.66%
Mortality in the general population (per 10 000 population)	0.4	5.8	12.0	17.0	9.1
Mortality in HCWs (per 10 000 population)	1.9	12.9	2.3	30.6	56.1
**Case fatality rate (March 1, 2020-Feb 28, 2021)**					
HCWs	0.98%	2.20%	0.47%	1.08%	2.43%
General population	1.76%	3.64%	2.69%	4.01%	3.09%

Note: SA denotes South Africa; WC denotes Western Cape; KZ denotes KwaZulu-Natal

Supplementary Table 2. Total economic burden and economic cost per SARS-CoV-2 infection among HCWs in international dollars (I$)

	Total economic loss (I$ million in 2020)			Cost per SARS-CoV-2 infection among HCWs (I$ in 2020)		
	Moderate-impact scenario (95% CIs)	Low-impact scenario	High-impact scenario	Moderate-impact scenario (95% CIs)	Low-impact scenario	High-impact scenario
Kenya	$280.82 ($153.58 - $466.38)	$87.79	$603.06	$83,404.31 ($45,613.99 - $138,515.49)	$26,073.19	$179,108.90
Eswatini	$42.13 ($35.49 - $51.36)	$30.02	$60.17	$92,792.71 ($78,186.26 - $113,149.44)	$66,133.47	$132,533.97
Colombia	$1,189.07 ($1,092.28 - $1,316.36)	$1,033.31	$1,425.21	$28,347.56 ($26,041.24 - $31,381.60)	$24,633.38	$33,976.21
SA-WC	$795.48 ($712.14 - $887.63)	$660.16	$938.65	$79,524.93 ($71,190.56 - $88,737.46)	$65,996.88	$93,836.96
SA-KZN	$1,283.09 ($1,188.92 - $1,390.76)	$1,111.15	$1,490.82	$80,631.47 ($74,715.01 - $87,397.84)	$69,827.40	$93,686.29

Note: SA denotes South Africa; WC denotes Western Cape; KZ denotes KwaZulu-Natal

Supplementary Table 3. Country profiles of study sites

	Kenya	Eswatini	Colombia	SA-WC	SA-KZN
Population size	+++	+	+++	++	++
HCW density	+	++	+++	++	++
COVID-19 incidence in general population	+	++	+++	+++	++
COVID-19 mortality rate for the general population	+	+++	++	+++	++
Share of infections in HCWs compared to general population	+++	++	+	+++	+++
Share of deaths due to COVID-19 in HCWs compared to general population	++	++	+	+	++

Note: the number of + indicates the relative level of each indicator, with "+++" being the highest and "+" being the lowest. SA denotes South Africa; WC denotes Western Cape; KZN denotes KwaZulu-Natal. The detailed information is presented in Supplemental Table 1.

Supplementary Table 4. Sources of key parameters for Kenya

Parameters	Value	Source
**General information**		
Population size in 2019	52,573,973	[1]
GDP per capita in 2019	1,817	[1]
GDP per capita growth rate in 2019	3.0%	[1]
Under 5 mortality rates in 2019	4.32%	[1]
Maternal mortality rate in 2019	0.34%	[1]
Birth rate (per 1000 population) in 2019	28.75	[1]
Number of admissions (per 1000 population) in 2018 (As a proxy for 2020)	35.0	[2]
Household size in 2019	3.9	[3]
Total health expenditure (USD in million) in 2020	5575	[1]
**Treatment costs per case**		
Mild-moderate covid-19 cases (home-based)	227	[4]
Mild-moderate covid-19 cases (facility-based)	844	[4]
Severe covid-19 cases	1,430	[4]
Critical cases	6,753	[4]
**Length of stay (for calculating the meal cost)**		
Mild-moderate covid-19 cases (facility-based)	1.0	Assumption
Severe covid-19 cases	7.0	[5]
Critical cases	10.3	[5]
**Other cost information**		
Travel cost per round trip for seeking care	14.17	[6]
Meal cost per day	3.18	[6]
GDP per capita per day	7.19	[1]
Monthly salary for doctors (USD)	1,448	[7]
Monthly salary for nurses (USD)	309	[7]
Monthly salary for clinical officers (USD)	407	[7]
Monthly salary for lab staff (USD)	241	[7]
Monthly salary for other staff (USD)	386	[7]
**Other parameters or intermediate parameters**		
Total number of HCWs	176,662	[8]
Exposure rate to HCWs	1.67%	Calculated
Odds ratio of SARS-CoV-2 infection due to exposure to HCWs	7.21	Calculated
Population attribution risk	9.40%	Calculated
HCWs SARS-CoV-2 infection rate	1.92%	Calculated
General population SARS-CoV-2 infection rate	0.20%	Calculated
Average age at deaths of COVID-19 cases among HCWs	50.8	Collected from MoH's Emergency Operation Center
Average age at deaths of COVID-19 cases among general population	55.0	Collected from MoH's Emergency Operation Center

Supplementary Table 5. Sources of key parameters for Eswatini

Parameters	Eswatini	Source
**General information**		
Population size in 2019	1,148,130	1
GDP per capita in 2019	3,895	1
GDP per capita growth rate in 2019	3.0%	1
Under 5 mortality rates in 2019	4.94%	1
Maternal mortality rate in 2019	0.44%	1
Birth rate (per 1000 population) in 2019	26.342	1
Number of admissions (per 1000 population) in 2010 (As a proxy for 2020)	64.5	9
Household size in 2019	5.84	3
Total health expenditure (USD in million) in 2020	323	1
**Treatment costs per case**		
Mild-moderate COVID-19 cases (home-based)	298	Estimated based on Kenya data
Mild-moderate COVID-19 cases (facility-based)	2,866	Estimated based Kenya data
Severe COVID-19 cases	4,844	Estimated from claims from health insurance agency
Critical cases	7,511	Estimated from claims from health insurance agency
**Length of stay (for calculating the meal cost)**		
Mild-moderate COVID-19 cases (facility-based)	1.0	Assumption
Severe covid-19 cases	22.5	10
Critical cases	8.8	10
**Other cost information**		
Travel cost per round trip for seeking care	10.0	11
Meal cost per day	5.0	11
GDP per capita per day	15.16	1
Monthly salary for doctors (USD)	7,715	Collected through a survey
Monthly salary for nurses (USD)	1,066	Collected through a survey
Monthly salary for other staff (USD)	525	Collected through a survey
**Other parameters or intermediate parameters**		
Total number of HCWs	7,726	Collected from the Ministry of Health
Exposure rate to HCWs	4.55%	Calculated
Odds ratio of SARS-CoV-2 infection due to exposure to HCWs	4.63	Calculated
Population attribution risk	14.20%	Calculated
HCWs SARS-CoV-2 infection rate	6.01%	Calculated
General population SARS-CoV-2 infection rate	1.56%	12
Average age at deaths of COVID-19 cases among HCWs	52	Collected from the Ministry of Health
Average age at deaths of COVID-19 cases among general population	59	Collected from the Ministry of Health

Supplementary Table 6. Sources of key parameters for Colombia

Parameters	Colombia	Source
**General information**		
Population size in 2019	50,339,443	[1]
GDP per capita in 2019	6,429	[1]
GDP per capita growth rate in 2019	1.9%	[1]
Under 5 mortality rates in 2019	1.38%	[1]
Maternal mortality rate in 2019	0.08%	[1]
Birth rate (per 1000 population) in 2019	14.882	[1]
Number of admissions (per 1000 population) in 2017 (As a proxy for 2020)	34.4	[13]
Household size in 2019	4.31	[3]
Total health expenditure (USD in million) in 2020	28,040	[1]
**Treatment costs per case**		
Mild-moderate COVID-19 cases (home-based)	283	Calculated based on Kenya data and [14]
Mild-moderate COVID-19 cases (facility-based)	2,441	Calculated based on Kenya data and [14]
Severe COVID-19 cases	4,126	[14]
Critical cases	8,022	[14]
**Length of stay (for calculating the meal cost)**		
Mild-moderate COVID-19 cases (facility-based)	1.0	Assumption
Severe COVID-19 cases	10.0	[15]
Critical cases	14.0	[15]
**Other cost information**		
Travel cost per round trip for seeking care	20.3	[16]
Meal cost per day	11.9	[16]
GDP per capita per day	25.19	[1]
Monthly salary for doctors (USD)	1,900	[17]
Monthly salary for nurses (USD)	917	[18]
Monthly salary for other staff (USD)	917	Assumption (to be the same as nurses)
**Other parameters or intermediate parameters**		
Total number of HCWs	838,651	MoH
Exposure rate to HCWs	6.20%	Calculated
Odds ratio of SARS-CoV-2 infection due to exposure to HCWs	1.32	Calculated
Population attribution risk	1.90%	Calculated
HCWs SARS-CoV-2 infection rate	5.00%	Calculated
General population SARS-CoV-2 infection rate	4.47%	[12]
Average age at deaths of COVID-19 cases among HCWs	54	Specific database on HCWs infection
Average age at deaths of COVID-19 cases among general population	54	Assumption (to be the same as HCWs)

Supplementary Table 7. Sources of key parameters for Western Cape and KwaZulu of South Africa

Parameters	SA-Western Cape	Source	SA-KwaZulu	Source
**General information**				
Population size	7,005,741	Collected	11,531,628	Collected
GDP per capita in 2019	6,001	[1]	6,001	[1]
GDP per capita growth rate in 2019	-1.2%	[1]	-1.2%	[1]
Under 5 mortality rate in 2019	2.33%	Collected from Department of Health, Western Cape	4.03%	Collected from Department of Health, KwaZulu
Maternal mortality rate in 2019	0.07%	Collected from Department of Health, Western Cape	0.08%	Collected from Department of Health, KwaZulu
Birth rate (per 1000 population) in 2019	20.51	[1]	20.51	[1]
Number of admissions (per 1000 population) in 2020	69.7	Collected from Department of Health, Western Cape	79.6	Collected from Department of Health, KwaZulu
Household size	4.32	[3]	6.14	[3]
Total health expenditure (USD in million) in 2020	4,032	Calculated based on population size and WHO database[19]	6,638	Calculated based on population size and WHO database[19]
**Treatment costs per case**				
Mild-moderate COVID-19 cases (home-based)	308	Calculated based on Kenya data and [10]	308	Calculated based on Kenya data and [10]
Mild-moderate COVID-19 cases (facility-based)	3,140	Calculated based on Kenya data and [10]	3,140	Calculated based on Kenya data and [10]
Severe COVID-19 cases	5,306	[10]	5,306	[10]
Critical cases	10,521	Calculated based on Kenya data and [10]	10,521	Calculated based on Kenya data and [10]
**Length of stay (for calculating the meal cost)**				
Mild-moderate COVID-19 cases (facility-based)	1.0	Assumption	1.0	Assumption
Severe COVID-19 cases	22.5	[10]	22.5	[10]
Critical cases	8.8	[10]	8.8	[10]
**Other cost information**				
Travel cost per round trip for seeking care	5.0	[20]	5.0	[20]
Meal cost per day	5.0	Assumption	5.0	Assumption
GDP per capita per day	22.81	[1]	22.81	[1]
Monthly salary for health care workers (USD)	7,715	Department of Health	7,715	Department of Health
**Other parameters or intermediate parameters**				
Total number of HCWs	35,317	Department of Health	68,862	Department of Health
Exposure rate to HCWs	3.07%	Calculated	4.46%	Calculated

Odds ratio of SARS-CoV-2 infection due to exposure to HCWs	6.25	Calculated	6.71	Calculated
Population attribution risk	13.90%	Calculated	20.30%	Calculated
HCWs SARS-CoV-2 infection rate	28.32%	Department of Health	23.11%	Department of Health
General population SARS-CoV-2 infection rate	4.24%	[12]	2.96%	[12]
Average age at deaths of COVID-19 cases among HCWs	56	Department of Health	56	Department of Health
Average age at deaths of COVID-19 cases among general population	63	Department of Health	63	Department of Health

Supplementary Table 8. Sources of parameters applied to all study sites

Items	Value	Source
Share of COVID-19 cases		
Mid-moderate	81%	[21]
Severe	14%	[21]
Critical	5%	[21]
Share of mid-moderate COVID-19 cases		
Home care	80%	Assumption
Facility-based care	20%	Assumption
Number of round trips per CIVID-19 cases	1.0	Assumption
Duration of being absent from the work		
Survived COVID-19 HCWs (days)	16.44	[22]
Deceased COVID-19 HCWs (years)	0.5	Assumption (mid-year death)
Reduction in productivity of other HCWs	10%	[23]
Contact intensity of inpatients compared to family members of HCW	20%	Assumption
Age at deaths		
Pregnant women	22.5	Assumption (mean age of pregnancy from one mid-income country)
children under 5	2.5	Assumption (death at mid-point between 0 and 5 years)
Odds ratio of SARS-CoV-2 infection/hospitalization of close contact of HCWs	1.79	[24]
Elasticity		
Healthcare worker elasticity of maternal mortality rate	-0.474	[25]
Healthcare worker elasticity of under 5 mortality rate	-0.231	[25]

Supplementary Fig. 1. Tornado graphs representing sensitivity analyses

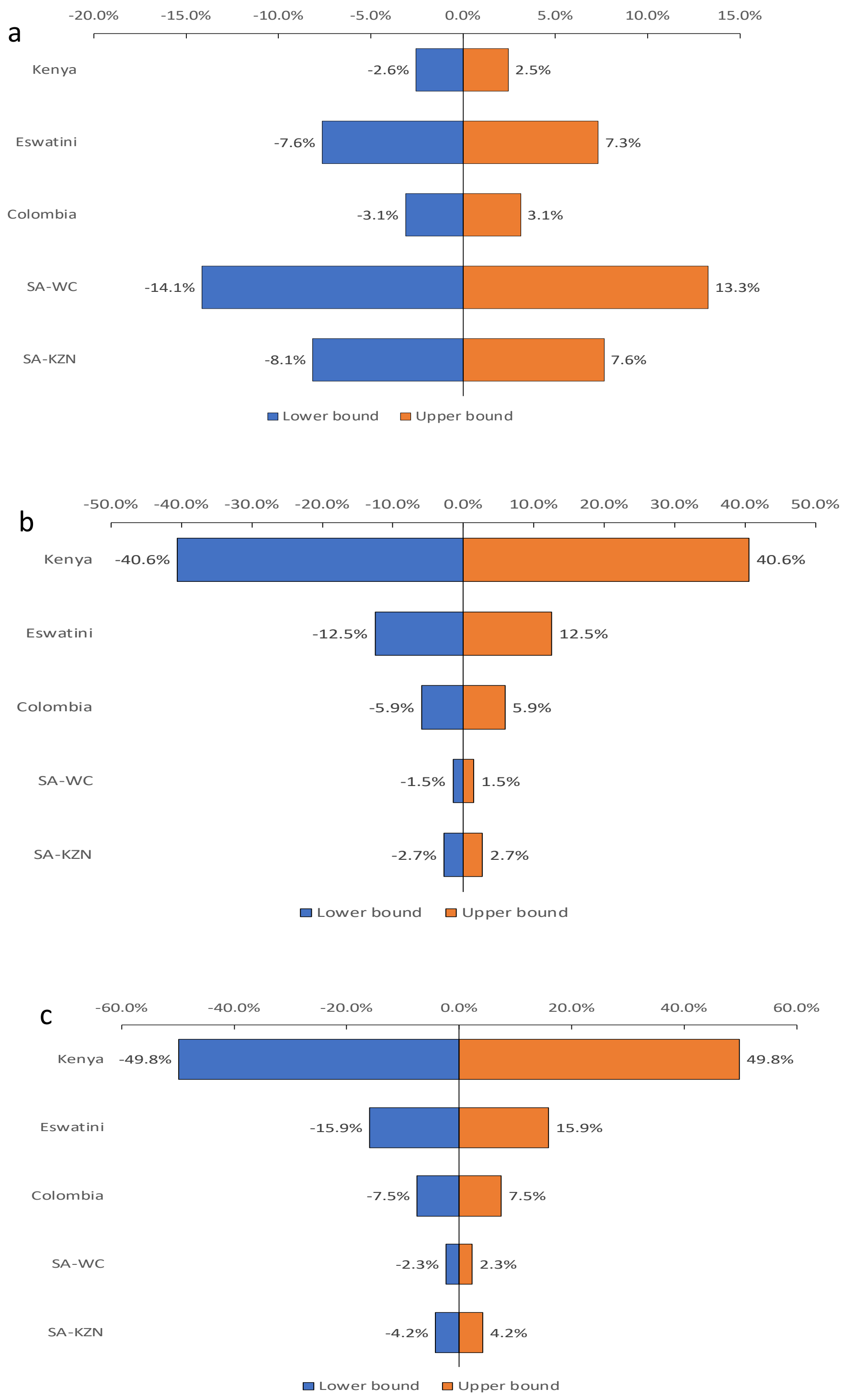

a Sensitivity analysis of the percentage of inpatients exposed to HCWs. b Sensitivity analysis of the productivity reduction among healthy HCWs. c Sensitivity analysis of HCW elasticity of U5MR and MMR. Note: SA denotes South Africa; WC denotes Western Cape; KZN denotes KwaZulu-Natal

Supplementary Methods. Estimation of exposure rate and odds ratio for secondary infection

To estimate E_i (the share of the population considered to be close contacts of HCWs for site *i*), we divided the sum of the average number of household contacts and patients admitted to health care facilities by the total population. To account for the difference in the duration and proximity of contact between HCWs and their household members compared to patients admitted to a health care facility, we assumed that inpatients had roughly 20% of the exposure to HCWs as household members.

$$E_i = \frac{(hosehold\ size_i - 1) * \#\ HCWs_i + \#hospital\ admissions_i * 20\%}{Total\ population\ size_i}$$

There is scarce literature on relative risk or odds ratio of SARS-CoV-2 infection due to HCW exposure. A national study in Scotland showed that family members of HCWs had a higher likelihood of SARS-CoV-2 infection, with an odds ratio of 1.79.[24] By assuming that the higher odds ratio of infection amongst close contacts of HCWs has a log-linear relationship with the ratio of SARS-CoV-2 infection in HCWs to the general population in any given country, we estimated the odds ratio for other countries based on Scotland's estimated odds ratio.

$$OR_i = 1 + 0.79 * \frac{\ln\left(\frac{Infection\ rate\ of\ HCW_i}{Infection\ rate\ of\ general\ population_i}\right)}{\ln\left(\frac{Infection\ rate\ of\ HCW_r}{Infection\ rate\ of\ general\ population_r}\right)}$$

where *i* refers to site *i*, and *r* refers to the reference country – Scotland.

Reference

1. World Bank. World Development Indicators. https://databank.worldbank.org/source/world-development-indicators (2022).
2. Dutta, A., Maina, T., Ginivan, M. & Koseki, S. Kenya health financing system assessment, 2018: Time to pick the best path. (Palladium, Washington, Dc, 2018).
3. Statista. Average household size in Kenya as of 2019, by county. https://www.statista.com/statistics/1225097/household-size-in-kenya-by-county/ (2022).
4. Barasa, E., *et al.* Examining unit costs for COVID-19 case management in Kenya. *medRxiv* (2020).
5. de Andrade, C.L.T., Pereira, C.C.A., Martins, M., Lima, S.M.L. & Portela, M.C. COVID-19 hospitalizations in Brazil's Unified Health System (SUS). *PLoS One* **15**, e0243126 (2020).
6. Vodicka, E.L., *et al.* Estimating the costs of HIV clinic integrated versus non-integrated treatment of pre-cancerous cervical lesions and costs of cervical cancer treatment in Kenya. *PLoS One* **14**, e0217331 (2019).
7. Kairu, A., *et al.* Cost of TB services in healthcare facilities in Kenya (No 3). *Int J Tuberc Lung Dis* **25**, 1028-1034 (2021).
8. Ministry of Health. Human resources for health dataset. (Ministry of Health, Kenya, Nairobi, Kenya, 2020).
9. Ministry of Health. Swaziland Annual Health Statistics Report. (Ministry of Health, Swaziland, Mbabane, Swaziland, 2010).
10. Cleary, S.M., Wilkinson, T., Tamandjou Tchuem, C.R., Docrat, S. & Solanki, G.C. Cost-effectiveness of intensive care for hospitalized COVID-19 patients: experience from South Africa. *BMC Health Serv Res* **21**, 82 (2021).
11. Ngcamphalala, C., Ostensson, E. & Ginindza, T.G. The economic burden of cervical cancer in Eswatini: Societal perspective. *PLoS One* **16**, e0250113 (2021).
12. Johns Hopkins University. COVID-19 Dashboard. https://coronavirus.jhu.edu/map.html (2021).
13. OECD. Health at a glance 2017 (OECD Publishing Paris, 2017).
14. elHospital. Tarifas de atencion en uci para pacientes con COVID-19. https://www.elhospital.com/temas/Tarifas-de-atencion-en-UCI-para-pacientes-con-COVID-19+134794 (2020).
15. Diaz, H., Espana, G., Castaneda, N., Rodriguez, L. & de la Hoz-Restrepo, F. Dynamical characteristics of the COVID-19 epidemic: Estimation from cases in Colombia. *Int J Infect Dis* **105**, 26-31 (2021).
16. Lee, J.S., *et al.* A multi-country study of the economic burden of dengue fever: Vietnam, Thailand, and Colombia. *PLoS Negl Trop Dis* **11**, e0006037 (2017).
17. Economic Research Institute. Medical doctor salary in Colombia. https://www.erieri.com/salary/job/medical-doctor/colombia (2021).
18. Salaryexplorer. Nurse average salary in Colomiba in 2020. https://www.salaryexplorer.com/salary-survey.php?loc=47&loctype=1&job=865&jobtype=3 (2020).
19. World Health Organization. Global Health Expenditure Database. https://apps.who.int/nha/database (2021).
20. Foster, N., *et al.* The economic burden of TB diagnosis and treatment in South Africa. *Soc Sci Med* **130**, 42-50 (2015).
21. Liu, D., *et al.* Risk factors for developing into critical COVID-19 patients in Wuhan, China: A multicenter, retrospective, cohort study. *EClinicalMedicine* **25**, 100471 (2020).

22. Faramarzi, A., Javan-Noughabi, J., Tabatabaee, S.S., Najafpoor, A.A. & Rezapour, A. The lost productivity cost of absenteeism due to COVID-19 in health care workers in Iran: a case study in the hospitals of Mashhad University of Medical Sciences. *BMC Health Serv Res* **21**, 1169 (2021).
23. Roberton, T., *et al.* Early estimates of the indirect effects of the COVID-19 pandemic on maternal and child mortality in low-income and middle-income countries: a modelling study. *Lancet Glob Health* **8**, e901-e908 (2020).
24. Shah, A.S.V., *et al.* Risk of hospital admission with coronavirus disease 2019 in healthcare workers and their households: nationwide linkage cohort study. *BMJ* **371**, m3582 (2020).
25. Anand, S. & Barnighausen, T. Human resources and health outcomes: cross-country econometric study. *Lancet* **364**, 1603-1609 (2004).
