# Supplementary material for: Modelling the economic burden of SARS-CoV-2 infection in health care workers in four countries
Source: Nat Commun. 2023 May 16;14:2791. doi: 10.1038/s41467-023-38477-7 (PMC10185455; doi:10.1038/s41467-023-38477-7)
Supplement: Supplementary file 3 — Reporting Summary [file 41467_2023_38477_MOESM3_ESM.pdf]

nature portfolio

Corresponding author(s): Wu Zeng

Last updated by author(s): Apr 25, 2023

Reporting Summary

Nature Portfolio wishes to improve the reproducibility of the work that we publish. This form provides structure for consistency and transparency in reporting. For further information on Nature Portfolio policies, see our Editorial Policies and the Editorial Policy Checklist.

Statistics

For all statistical analyses, confirm that the following items are present in the figure legend, table legend, main text, or Methods section.

n/a	Confirmed	
☒	☐	The exact sample size (*n*) for each experimental group/condition, given as a discrete number and unit of measurement
☒	☐	A statement on whether measurements were taken from distinct samples or whether the same sample was measured repeatedly
☒	☐	The statistical test(s) used AND whether they are one- or two-sided<br>*Only common tests should be described solely by name; describe more complex techniques in the Methods section.*
☒	☐	A description of all covariates tested
☒	☐	A description of any assumptions or corrections, such as tests of normality and adjustment for multiple comparisons
☐	☒	A full description of the statistical parameters including central tendency (e.g. means) or other basic estimates (e.g. regression coefficient) AND variation (e.g. standard deviation) or associated estimates of uncertainty (e.g. confidence intervals)
☒	☐	For null hypothesis testing, the test statistic (e.g. *F*, *t*, *r*) with confidence intervals, effect sizes, degrees of freedom and *P* value noted<br>*Give P values as exact values whenever suitable.*
☒	☐	For Bayesian analysis, information on the choice of priors and Markov chain Monte Carlo settings
☒	☐	For hierarchical and complex designs, identification of the appropriate level for tests and full reporting of outcomes
☒	☐	Estimates of effect sizes (e.g. Cohen's *d*, Pearson's *r*), indicating how they were calculated

Our web collection on statistics for biologists contains articles on many of the points above.

Software and code

Policy information about availability of computer code

Data collection	No software was used to collect data
Data analysis	All the simulations and analyses were performed using R (version 4.2.2). The code is available at https://github.com/wuzengcn/HRH and https://doi.org/10.5281/zenodo.7856086.

For manuscripts utilizing custom algorithms or software that are central to the research but not yet described in published literature, software must be made available to editors and reviewers. We strongly encourage code deposition in a community repository (e.g. GitHub). See the Nature Portfolio guidelines for submitting code & software for further information.

Data

Policy information about availability of data

All manuscripts must include a data availability statement. This statement should provide the following information, where applicable:

- Accession codes, unique identifiers, or web links for publicly available datasets
- A description of any restrictions on data availability
- For clinical datasets or third party data, please ensure that the statement adheres to our policy

The data were provided in the appendices associated with the article. All the parameters used for the simulation are also available at https://github.com/wuzengcn/HRH and https://doi.org/10.5281/zenodo.7856086. Data from the World Develop Indicators and Johns Hopkins University's COVID-19 dashboard are publically accessible.

Human research participants

Policy information about studies involving human research participants and Sex and Gender in Research.

Reporting on sex and gender	NA
Population characteristics	NA
Recruitment	NA
Ethics oversight	NA

Note that full information on the approval of the study protocol must also be provided in the manuscript.

Field-specific reporting

Please select the one below that is the best fit for your research. If you are not sure, read the appropriate sections before making your selection.

☐ Life sciences ☒ Behavioural & social sciences ☐ Ecological, evolutionary & environmental sciences

For a reference copy of the document with all sections, see nature.com/documents/nr-reporting-summary-flat.pdf

Behavioural & social sciences study design

All studies must disclose on these points even when the disclosure is negative.

Study description	This is a modelling study using secondary data
Research sample	All the data used in this study were either obtained at the national level through census or from publically available country level data (e.g. GDP/capita) or literature. No sampling was conducted.
Sampling strategy	This study used aggregated national level data to perform the analysis. No sampling was conducted.
Data collection	We used a mix of data sources: a) primary data collection, in which the World Bank country offices obtained and verified data provided by national authorities on SARS-CoV-2 infection in HCWs and their outcomes (i.e., survival or death) in the first year of the pandemic, as well as average HCW income by profession to the extent possible; b) World Development Indicators on country demographics, macroeconomic figures (e.g., GDP per capita) and health sector indicators (e.g., total health expenditure, total government health expenditure, hospital admission rates, under-five mortality rate, maternal mortality rate); c) the Johns Hopkins University database on SARS-CoV-2 infections and deaths in the general population; d) peer-reviewed journal publications or grey literature for other key parameters such as treatment cost per case, the composition of cases by severity level, length of stay in a medical facility, duration of absence from work; e) the research team's own assumptions; and f) parameter extrapolation from known sources
Timing	March 2020-Feb 2021
Data exclusions	No data were excluded
Non-participation	No participants were involved in this study
Randomization	This study is not a randomized trial, and thus randomization was not applied.

Reporting for specific materials, systems and methods

We require information from authors about some types of materials, experimental systems and methods used in many studies. Here, indicate whether each material, system or method listed is relevant to your study. If you are not sure if a list item applies to your research, read the appropriate section before selecting a response.

Materials & experimental systems

n/a	Involved in the study
☒	☐ Antibodies
☒	☐ Eukaryotic cell lines
☒	☐ Palaeontology and archaeology
☒	☐ Animals and other organisms
☒	☐ Clinical data
☒	☐ Dual use research of concern

Methods

n/a	Involved in the study
☒	☐ ChIP-seq
☒	☐ Flow cytometry
☒	☐ MRI-based neuroimaging
